# Detection of Mild Cognitive Impairment from Language Markers with Crossmodal Augmentation

**Published:** 2023

**Authors:** Guangliang Liu, Zhiyu Xue, Liang Zhan, Hiroko H. Dodge, Jiayu Zhou

**Affiliations:** 1 Department of Computer Science and Engineering, Michigan State University, East Lansing, MI 48824, USA; 2 Department of Electrical & Computer Engineering, University of Pittsburgh, Pittsburgh, PA 15261, USA; 3 Department of Neurology, Massachusetts General Hospital, Harvard Medical School, Boston, MA 02129, USA; Department of Neurology, Layton Aging and Alzheimer’s Disease Center, Oregon Health & Science University, Portland, OR 97239, USA

**Keywords:** Mild Cognitive Impairment, Multi-modality Analysis, Crossmodal Augmentation

## Abstract

Mild cognitive impairment is the prodromal stage of Alzheimer’s disease. Its detection has been a critical task for establishing cohort studies and developing therapeutic interventions for Alzheimer’s. Various types of markers have been developed for detection. For example, imaging markers from neuroimaging have shown great sensitivity, while its cost is still prohibitive for large-scale screening of early dementia. Recent advances from digital biomarkers, such as language markers, have provided an accessible and affordable alternative. While imaging markers give anatomical descriptions of the brain, language markers capture the behavior characteristics of early dementia subjects. Such differences suggest the benefits of auxiliary information from the imaging modality to improve the predictive power of unimodal predictive models based on language markers alone. However, one significant barrier to the joint analysis is that in typical cohorts, there are only very limited subjects that have both imaging and language modalities. To tackle this challenge, in this paper, we develop a novel crossmodal augmentation tool, which leverages auxiliary imaging information to improve the feature space of language markers so that a subject with only language markers can benefit from imaging information through the augmentation. Our experimental results show that the multi-modal predictive model trained with language markers and auxiliary imaging information significantly outperforms unimodal predictive models.

## 1. Introduction

Alzheimer’s disease is the fifth-leading cause of death among individuals at age 65 and older.^1^ A person with Alzheimer’s will live through years of morbidity during the disease progression. Mild cognitive impairment (MCI) is the prodromal stage of Alzheimer’s disease and serves as an important stage for early intervention and subject recruitment of cohort studies for understanding the disease and developing novel treatments.

There are extensive efforts on the identification of MCI and the associated markers. Because the progression of the disease is associated with structural changes in the brain,^2^ the potential of detection from brain imaging of various types has been widely studied. Especially, magnetic resonance imaging (MRI) has provided a non-invasive way of examining the structure of the brain and tracking its changes. Studies have associated measurements from MRI with early-stage dementia.^3, 4^ The availability of a large amount of MRI data from Alzheimer’s Disease Neuroimaging Initiative^5^ largely facilitated the development of machine learning algorithms for detection.^6–8^ Even though imaging markers from MRI are considered to be sensitive to early-stage MCI, the cost of MRI scans prevents them from being widely used for large-scale screening. The recent development of digital biomarkers, especially language markers, has shown promising sensitivity to detection of MCI.^9–14^ For example, language markers can be used in conversational agents deployed on mobile devices or smart speakers to obtain a risk assessment of MCI.^9^ However, the investigation of language markers is still in the early phase, where a critical issue is that the cohort sizes for studying language markers remain very limited,^15^ demanding more data to unleash their power.

While imaging markers give anatomical descriptions of the brain, language markers capture the behavior characteristics of early dementia subjects. Such differences suggest the benefits of multi-modality analysis, where auxiliary information from the imaging modality can improve the power of accessible language markers further. However, one significant barrier to the multi-modality joint analysis is that in typical cohorts, there are only very limited subjects that have both imaging and language modalities. For example, in a cohort study from the I-CONECT clinical trial,^15^ there are 40 subjects randomized for the experimental group for whom language makers (semi-structured conversations) are available. Yet among these subjects, there are only 16 subjects who have MRI scans available in the National Alzheimer’s Coordinating Center (NACC) medical records. Typical multi-modality analysis approaches often require a substantial amount of data points that are shared or “aligned” across modalities to calibrate across different modalities and seek a common subspace,^16^ and yet very few subjects in these cohorts can be used for existing multi-modality analysis. This results in a huge waste of collected data and often sub-optimal model performance due to insufficient sample size.

To tackle this challenge, in this paper, we developed a novel crossmodal augmentation tool, which leverages auxiliary imaging data to improve predictive modeling of language markers. Specifically, based on the language markers of a subject, the augmentation model constructs a feature embedding from the imaging domain by gauging its similarity with respect to other subjects and relating to the interconnection between two modalities. To achieve this, we introduced a model that learns to measure the consistency between any pair of language features and imaging features. The design of our model gives high sample efficiency, so that the learning can be done even when there are only a few subjects that have both modalities. During inference, the model assigns weights of existing imaging embedding for a given language embedding to construct the augmented features. We show in our empirical study that the proposed early MCI detection model, by augmenting language modality with constructed features from imaging information, significantly outperforms unimodal models and straightforward multi-modality models using aligned multi-modal data alone.

## 2. Related Works

### Early Detection of MCI.

Early detection of MCI is of great clinical importance and predictive models are built from a variety of data types, such as clinical information,^16^ neuroimaging,^4, 17^ and, more recently, digital biomarkers.^12^ Neuroimaging captures the structural information of the brain, and therefore imaging markers, especially from structural MRI,^17^ have shown great sensitivity. Besides being non-intrusive, the cost of imaging markers is still prohibitive for large-scale screening of early dementia. Recent advances in digital biomarkers,^18^ such as language markers, have provided an accessible and affordable alternative.^12^ From the spontaneous speech, we can extract linguistic features (e.g., word preference, syntactic features, semantic features, data-driven word embedding) and acoustic information (e.g., MFCC).^11, 12^ It has been recently shown that combining acoustic features and linguistic features delivers an improved prediction performance.^12, 13^ The development of language markers is still in the very early phase, with limited data available for modeling. The analysis can benefit from more data from different data sources to deliver high predictive performance.

### Multi-modality Learning.

Multi-modality learning aims to characterize a concept (such as MCI) from different perspectives by using the complementary features from different modalities.^19^ The paradigm has been widely used in biomedical and bioinformatics studies due to the ubiquitous need for joint analysis on multiple data modalities. Early fusion approaches fuse the features in the data/feature space and train a machine learning model based on the fused features. Late fusion approaches build independent models associated with an individual modality and produce the final classification score by combining the outcomes from each model. Most existing multi-modality approaches require the majority of data to be aligned across different modalities to learn the underlying connections among the modalities, which is the motivation of this work.

### Feature Synthesis.

Linear combination has been widely used in data analysis for synthesizing samples. SMOTE-based methods^20, 21^ alleviate the class imbalance problem by manually synthesizing new samples with linear combination in data space or feature space. Linear combination with Gaussian weights^22^ is used to generate samples for biometrics tasks. More recently, MixUp-based methods^23, 24^ augment the training data using synthesized samples generated by linear combination, increasing performance and enhancing robustness.^25^ Linearly synthesized T1 MRI features are shown to facilitate accurate attenuation correction maps.^26^ We adopt linear combinations to construct features due to performance and computational efficiency.

## 3. Methods

### 3.1. Data

We use conversational data and imaging data from an ongoing clinical trial I-CONECT (Clinicaltrials.gov: NCT02871921)^a^. Briefly, this trial examines whether frequent conversational engagement through video chats with standardized interviewers improves cognitive functions. Only the experimental group engages in frequent semi-structured conversations while the control group receives only 10 minutes of phone check-ins weekly. The recorded semi-structured conversation used among the experimental group (N=40) was utilized in the current analyses. Among these 40 subjects with available language markers, half of them are MCI, and the rest are cognitively normal (NL). For each subject, we randomly sample 15 individual conversational recordings and employ automatic speech recognition (ASR) to generate transcripts. Only the subjects’ responses are used for analysis, the linguistic features are extracted over a whole transcript. Therefore there are 120 linguistic feature vectors as elaborated in the next subsection. For imaging data, we use the structural MRI data of 43 subjects from I-CONECT, where 26 of them are MCI, and 17 of them are cognitively normal. We extract variables from the T1-weighted (T1w) MRI data and diffusion MRI (dMRI) of each subject, and follow our previous work^17^ to extract corresponding imaging features. Specifically, from T1w MRI we used the cortical volume and thickness measurements for 74 brain region-of-interests (ROIs) extracted by FreeSurfer. From dMRI we derived brain connectome network over 85 ROIs using Probtrackx tractography, following the protocol in Ref. 17. For each subject, we extracted the fiber counting feature among 85 ROIs. 16 subjects have both conversational recordings and MRI data, the others only have either imaging data or conversational data. And all subjects have clinical diagnoses (MCI or NL), which are determined according to the agreement of neurologists and neuropsychologists by referring to publicly available diagnostic criteria.^27^

### 3.2. Language and Imaging Markers for Early Detection of MCI

From raw speech data, we first translate the subjects’ responses into text using Google ASR. From the text, we extract a comprehensive set of linguistic features from various levels of lexicon, syntax, and semantics. All features are extracted over the whole transcript. One example of lexical features is the average word length which measures the average number of letters to form a word. Syntactic features indicate how complex the syntactic structure of a sentence is. For example, the depth of syntactic tree counts the depth of a constituent syntax tree.^28^ In terms of semantic features, we considered two kinds of features: local coherence and global coherence. Local coherence measures how the semantics of sentences change within the subject’s responses to a question. We employ fasttext^29^ to get the embedding representation of a sentence and calculate the cosine similarity between any two connective sentences. For the imaging data, we consider both T1w features and brain network features.^30^ Mean/varaince statistics is available for all features, except those of LIWC word category and dMRI fiber count, are available. Because that the number of features is much larger than the sample size, which may easily lead to overfitting. We select features by stability selection,^17^ 56 imaging features and 112 language features are reserved.

### 3.3. Leverage Auxiliary Imaging Information in MCI Detection from Language Markers

The goal of this paper is to augment the feature space of language markers utilizing complimentary information from the auxiliary imaging modality, and ultimately improve the predictive performance. In this way, all subjects with only accessible language markers in the future can benefit from performance improvements.

The early MCI detection from language data is formulated as a classification problem,^12^ using language markers from a subject to predict the subject’s clinical label. The proposed solution leverages the entire data available for training. The training data *D*_train_ includes a set of subjects that have language markers *D*_lang_ and another set of subjects that have imaging markers *D*_img_. There are overlapped subjects that have both modalities, denoted by *D*_align_, i.e., *D*_train_ = {*D*_lang_ ∪ *D*_img_ ∪ *D*_align_}. A sample (*X*_lang_, *X*_img_, *Y*) ∈ *D*_align_ has language markers *X*_lang_ and imaging markers *X*_img_, and *Y* ∈ {0, 1} is the clinical label such that 1 is MCI and 0 is cognitive normal (NL). We use the multi-modality training data *D*_train_ to learn a crossmodal augmentation model *g*_*ω*_, parameterized by *ω*. Given any set of language markers $x_{\text{lang}}\in {\mathbb{R}}^{112}$, the model generates an augmented feature vector *x*_aug_ that has the same dimension as the imaging markers (56 in our study). We then train a classifier *f*, parameterized by *θ*, that takes the augmented features [*x*_lang_, *x*_aug_] to predict the clinical label.

### 3.4. Crossmodal Augmentation Model

The key idea of the crossmodal augmentation model is to build a prediction model *g*_*ω*_: given two modality vectors for a subject, one from imaging and one from language, the model *g*_*ω*_ predicts whether the two modality vectors are from the same subject. The foundation of the augmentation model has the same spirit as other multi-modality models, that is to capture the underlying connection between the pair modalities. During the inference, when the subject has only language modality (*x*_lang_, *y*), the model *g*_*ω*_ is then used to assign weights to all available imaging feature vectors (from other subjects) to construct an augmented feature vector from *k*–highest predicted imaging features. The proposed crossmodal augmentation model can be extended to more than two modalities, and we leave the methodology extensions and their theoretical analysis to an extended version of this work.

The paired design allows us to construct a training dataset $D_{\text{learning}}^{\prime }$ for crossmodal augmentation model, which is the key to our sample efficiency. For each sample with both imaging and language features (*x*_lang_, *x*_img_, *y*) in *D*_align_, we randomly sample an image feature $d_{\text{img}}=(x_{\text{img}}^{\prime },y^{\prime })\in D_{\text{img}}$ with the constraint that the label of *D*_img_ is different from that of *d*_align_, to ensure that data modalities in manually created samples are not aligned. On the contrary, we create the aligned samples by randomly sampling imaging features with the same label to *x*_*lang*_. The procedure creates two new samples (*x*_lang_, *x*_img_, 1) and $\left(x_{\text{lang}},x_{\text{img}}^{\prime },0\right)$, where label 1 means aligned and 0 otherwise, to train the crossmodal augmentation model *g*_*ω*_. Then a augmented training dataset $D_{\text{aug}}^{\prime }$ for classification model *f*_*θ*_ can be constructed by *g*_*ω*_. Algorithm 1 summarizes the training procedure including the training the proposed crossmodal augmentation model *g*_*ω*_ and MCI detection model *f*_*θ*_.

## 4. Experimental Results and Analysis

### 4.1. Experimental settings

In the experiment, we use the data of 83 subjects, where 40 of them have conversational recordings, 43 of them have imaging data, and only 16 subjects have both conversational recordings and imaging data. For each subject with conversational recordings, there are 15 transcripts used for data efficiency. For each experiment, we randomly sample 4 MCI subjects and 4 NL subjects from the 16 subjects with both data modalities as test data. We consider 100 different random train-test splits for each model and report the mean Area under the ROC curve (AUC), Accuracy, and F1 score on the test data. We adopt the elastic net regularized logistic regression^31^ as our MCI detection model and employ a gradient-boosting decision tree as the crossmodal alignment model, with both implemented by the Python library scikit-learn.^32^ To mitigate the influence of incorrect prediction from the crossmodal alignment model, we pick up a large number of subjects, e.g. 15, for imaging feature synthesis.


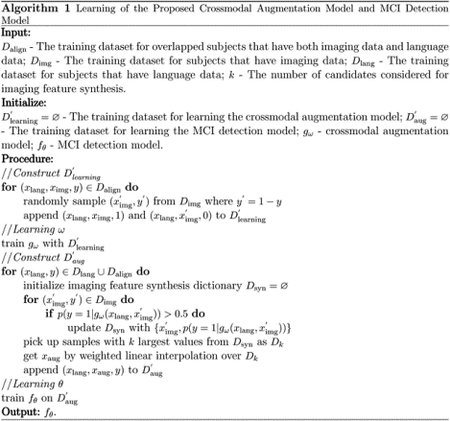


Our main goal is to augment language markers using imaging information and therefore evaluate the predictive performance of models learned with the augmented marker space (Ours-Lang-AugImg). We also investigate a less practical setting, i.e., augmenting imaging markers using language information (Ours-Img-AugLang). We implement baseline models trained with only one data modality, the MCI-Lang model adopts language data and the MCI-Img model is learned with imaging data. The source code and experiment scripts are available at https://github.com/illidanlab/XModalAug.

### 4.2. MCI Detection using Crossmodal Augmentation

The MCI detection performance of baseline unimodality approaches and two crossmodal augmentation approaches is shown in Table 1. a) We see that for unimodality prediction settings, MCI-Img delivered an exceptional performance of 0.97 AUC. This confirms the power of neuroimaging. b) MCI-Lang yields an average of 0.8 AUC, showing the promise of the accessible digital biomarker. c) With the augmented variables from auxiliary imaging information, Ours-Lang-AugImg receives a striking performance gain to an AUC of 0.97, significantly outperforming the MCI-Lang and slightly outperforming MCI-Img. d) The best performer is Ours-Img-AugLang which uses the imaging markers as the main predictor, treats language markers are auxiliary information, and uses them to create augmented variables. The model has less practical usage due to the lack of accessibility of imaging markers, but the results confirm the benefits of joint analysis of imaging and language markers.

### 4.3. Straightforward Multi-modal Model using Aligned Multi-modal Data

In this section, we validate straightforward multi-modal prediction methods based on the small amount of aligned multi-modal dataset to show that our crossmodal augmentation method can effectively utilize large-sized partially-aligned multi-modal data. To fully explore the predictive power of multi-modal data, we implemented various multi-modal fusion methods: *ConFusion* concatenates imaging feature vector and language feature vectors, then feed the concatenated feature vector to the MCI detection model. *VotingAvgFusion* generates the mean prediction score of two individual classification models trained with language data and imaging data, respectively. *InterFusion* implements outer product operations on the language feature vector and the imaging feature vector. *InterConFusion* is a mix of ConFusion and InterFusion by concatenating the outer product of two feature vectors and the original feature vectors.

The performance of multi-modality fusion methods is shown in Table 2. ConFusion is the best performer. A possible hypothesis behind the results is that, since language markers are weaker predictors than imaging markers, and non-linear fusion methods (VotingAvgFusion, InterFusion and InterConFusion) may introduce noise to the imaging markers.

### 4.4. Top Language Markers and Imaging Markers in Predictive Models

We investigate important language and imaging markers identified by the predictive model, and also how the augmentation impacts these top markers in the model. On the language marker side, we extract the coefficients of our best MCI detection model trained with language data and calculate the feature importance by the absolute value of coefficients. The top 10 important language markers are listed in the first sub-table of Table 3. MCI subjects prefer personal pronouns like “we”, “you”, “I”, but NL subjects take words related to space. An interesting finding is that MCI subjects tend to use long phrases, but NL subjects often prefer long verb phrases. The syntactic feature “coexistence of adverb phrase, verb phrase, and noun phrase” has the highest importance, which means a single sentence contains at least one adverbial phrase, one verb phrase, and one noun phrase. Constructing a sentence with a complex syntactic structure can be more challenging for MCI subjects, which is also shown by previous study.^33^ Moreover, the word length is effective in detecting MCI since MCI subjects are more likely to use words containing fewer letters. Also, MCI subjects’ expressions are not as coherent as those of NL subjects. The middle section of Table 3 shows top imaging markers extracted from the MCI detection model trained with imaging data. The feature name column represents a particular attribute of a given brain region, and the function column highlights the specific function of that brain region. We see that top-ranked feature variables are exclusively from T1-weighted MRI.

After applying crossmodal augmentation, we now have a set of auxiliary variables available, in addition to the original language markers we input to the augmentation model. Note that the augmented variables have one-one correspondence to imaging markers, and yet they do not necessarily possess the meaning. In this section, we show how top-ranked feature variables changed in the predictive models after using the augmented feature space. The bottom section of Table 3 shows the top markers in the model using augmented language markers. We see that 1) the top-ranked features are dominated by auxiliary variables from our crossmodal augmentation model, demonstrating the importance and effectiveness of the proposed augmentation scheme, even though these markers are in fact generated according to the guidance of language markers. 2) the top-ranked augmented features and top-ranked imaging markers in the middle section of Table 3 are not consistent. Since the augmentation tries to synthesize imaging markers from language markers, the inconsistency in ranking means that not all imaging markers can be well synthesized through the linear combination, under the guidance of language markers. Some of the imaging variables may be better augmented by language markers due to their implicit connections to language functionalities.^34^ 3) there are two dMRI features in top-ranked augmented features, whereas the corresponding actual imaging markers do not stand out in the imaging unimodal learning. This directly shows the importance of diffusion MRI variables in the crossmodal analysis and their differential benefits in modeling MCI, as also suggested in our previous work.^17^

## 5. Discussion

In this study, we propose a crossmodal augmentation method to augment language markers with synthesized variables guided by auxiliary imaging data, for improved performance on MCI detection. Our augmentation model learns to efficiently associate language information and imaging information with only a limited number of subjects having both data modalities. The learned model will then use the language markers of a subject to construct auxiliary variables by a linear combination of imaging markers from those that possess imaging information. The augmented language markers significantly improve the AUC score of MCI prediction from 0.8 to 0.973. We also validate the generalization of our method by augmenting imaging markers with language features, which contributes to an AUC score of 0.98. Our method tackles the problem of joint analysis to multi-modal data with limited crossmodal alignment supervision.

Though the proposed crossmodal augmentation approach has shown exceptional performance improvements, there are future studies and further improvements remain. 1) First of all, the augmented variables are constructed by a linear combination of a set of given imaging markers or “anchor” imaging markers. Such dependency has motivated us to study the impact of the anchor makers later on, with the possibility of using refined anchor markers. 2) Second, due to the small sample size available for training, we used the restricted assumption that the feature space of imaging data is linear, which may be further improved by non-linear assumptions. 3) Our analysis has shown a deeply convoluted relationship between language markers and imaging markers, as suggested by the top-ranked features. Such a relationship and its implications need further analysis, the understanding of which can further guide our improvements on the augmentation. 4) Last but not least, we only validate the crossmodal augmentation over two modalities. With the high sample-efficiency design, we can directly extend the approach to more than two modalities, and we will investigate these scenarios in our future work.

The proposed method can be directly extended to various clinical applications. One example is to improve MCI detection performance given only dialogue data. Assume that only the easily acquired dialogue data and public MRI data^5^ are available in the institution A. One can learn a crossmodal alignment model with a private and labeled dataset from the institution B, and this dataset includes aligned dialogue and MRI data. Then apply the crossmodal alignment model to the dataset of A through considering the domain shift between two MRI datasets. Since the private MRI data from B is not released, we can achieve privacy-preserving prediction in the condition of missing modality. We leave this discussion to our future work.

## Figures and Tables

**Table 1.** MCI detection performance. Our method employs both data modalities and outperforms baseline models trained with only one data modality.

Models	Train Data Size	AUC	Accuracy	F1
MCI-Lang MCI-Img	32 subjects 35 subjects	0.80 ± 0.01 0.969 ± 1*e*^−6^	0.73 ± 0.07 0.846 ± 1*e*^−11^	0.71 ± 0.01 0.872 ± 1*e*^−11^
Ours-Lang-AugImg	32 subjects	0.973 ± 0.001	0.848 ± 0.008	0.873 ± 0.004
**Ours-Img-AugLang**	35 subjects	0.98 ± 0.002	0.87 ± 0.005	0.89 ± 0.005

**Table 2.** The straightforward multi-modal MCI prediction with different multi-modality fusion methods based on linguistic features and imaging features. The ConFusion method that does not apply any fusion strategy outperforms other multi-modal fusion methods.

Models	Train Data Size	AUC	Accuracy	F1
VotingAvgFusion	8 subjects	0.51 ± 0.17	0.52 ± 0.15	0.63 ± 0.07
InterFusion	8 subjects	0.62 ± 0.05	0.56 ± 0.09	0.64 ± 0.07
InterConFusion	8 subjects	0.82 ± 0.08	0.71 ± 0.09	0.74 ± 0.08
ConFusion	8 subjects	0.84 ± 0.015	0.77 ± 0.009	0.78 ± 0.005

**Table 3.** High-impact feature variables in predictive models. Note that the prefix AUG means augmented feature variables from the Ours-Lang-AugImg model, the names after AUG show the correspondent feature names in imaging marker but they are not actual imaging features. coeff represents the logistic regression coefficients, higher absolute value of coeff indicates the associated feature is more important.

Top-ranked features from predictive model using only language markers		
Feature name		coeff
coexistence of adverb phrase, verb phrase and noun phrase		−2.04
word length in letters		−1.05
LIWC word category of nonfluencies		−0.91
LIWC word category of we		0.85
LIWC word category of anger		−0.68
LIWC word category of space		−0.66
verb phrase span ratio		−0.64
average phrase span		0.59
LIWC word category of sexual		−0.55
global coherence		0.53
Top-ranked features from predictive model using only imaging markers		
Feature name	Function	—coeff—
thickness of left lateral orbito frontal	Emotion	0.50
cortical volume of left pars orbitailis	Language	0.42
thickness of left posterior cingulate cortex	Neural Communication	0.42
cortical volume of left inferior temporal	Vision	0.41
cortical volume of left supramarginal gyrus	Language	0.33
thickness of right peri calcarine	Vision	0.30
thickness of right cauda lmiddle frontal	Memory	0.29
thickness of left posterior cingulate cortex	Neural Communication	0.28
cortical volume of right inferior temporal	Vision	0.27
thickness of left fusiform	Neural Communication	0.26
Top-ranked features from Ours-Language-AugImg		
Feature name	Function	—coeff—
AUG: cortical volume of left pars orbitalis	Language	1.1
AUG: cortical volume of right supramarginal	Language	1.07
AUG: thickness of left lateral orbito frontal	Emotion	1.05
AUG: thickness of left posterior cingulate	Neural Communication	0.97
AUG: cortical volume of left inferior temporal	Vision	0.82
AUG: thickness of left posterior cingulate	Vision	0.78
AUG: thickness of left caudal middle frontal	Memory	0.68
AUG: dMRI: fiber count right bankssts	Language/Biological perception	0.68
AUG: dMRI: fiber count left caudal middle frontal	Memory	0.65
AUG: cortical volume of left isthmus cingulate	Emotion	0.62

## Footnotes

a The data is available upon request at https://www.i-conect.org/.
